# Left atrioventricular remodeling in the assessment of the left ventricle diastolic function in patients with heart failure: a review of the currently studied echocardiographic variables

**DOI:** 10.1186/1476-7120-6-56

**Published:** 2008-11-16

**Authors:** Luiz C Danzmann, Luiz Carlos Bodanese, Ilmar Köhler, Marco R Torres

**Affiliations:** 1 Universidade Luterana do Brasil, Porto Alegre, Brazil; 2 Pontifícia Universidade Católica do Rio Grande do Sul, Porto Alegre, Brazil; 3 Universidade Federal do Rio Grande do Sul, Porto Alegre, Brazil

## Abstract

Multiparametric echocardiographic imaging of the failing heart is now increasingly used and useful in decision making in heart failure. The reasons for this, relies on the need of different strategies of handling these patients, as differentiation of systolic or diastolic dysfunction, as well as on the gamma of approaches available, such as percutaneous and surgical revascularization, devices implantations, and valvular regurgitations and stenosis corrections. Congestive heart failure in patients with normal left ventricular diameters or preserved left ventricular ejection fraction had been pointed out recently as present in a proportion so high as 40 to 50 percent of cases of heart failure, mainly due to the epidemics in well developed countries, as is the problem of not well controlled metabolic states (such as obesity and diabetes), but also due to the *real word *in developing countries, as is the case of hypertension epidemics and its lack of adequate control. As a matter of public utility, the guidelines in the diagnosis and treatment of such patients will have to be cheap, available, easily reproducible, and ideally will furnish answers for the clinician questions not in a binary "black or white" manner, but with graduations, so if possible it has to be quantitative. The present paper aim to focus on the current clinical applications of tissue Doppler and of left atrial function and remodeling, and its pathophysiologic relationship with the left ventricle, as will be cleared in the documented review of echocardiography that follows, considering that the need of universal data on the syndrome of the failing heart does not mean, unfortunately, that all patients and clinicians in developing countries have at their own health facilities the same imaging tools, since they are, as a general rule, expensive.

## Heart failure and its epidemiological importance

Heart failure is considered a world endemic problem and data ratified by The European Society of Cardiology (ESC), according to recent publications, estimates a prevalence of symptomatic manifestations in the general European population ranging from 0.4 to 2% [1]. In other countries, for over three decades, acute myocardial infarction (AMI) is the most frequent cause of deaths among the adult population – as, for instances, seen in southern Brazilian cities-, and mortality exhibits increase for both sexes as the age increases [2]. Cardiac failure (CF) in fact is a complex syndrome, embracing systemic complications determined by all forms of heart disease, and it is the common end for the most prevalent illnesses, like the atherosclerotic coronary disease and systemic arterial hypertension (SAH). The population percentage growth of elderly people in Brazil is a fact, at it is estimated to be around 30 million over 60 years of age in 2025, or 15% of the expected Brazilian population for the period [3], and this will much contribute for the increase of incidence and prevalence in the rates of heart failure. In this context, the epidemiological importance of the syndrome justifies the significant growing interest in the research area through the organization of CF specific investigation centers in University hospitals, and public policies of investment aiming at primary or secondary prevention of the risky population.

## Heart structure redefinition and function importance in the cardiac failure study

Under the physiopathologic point of view, CF is characterized as myocardial function failure. Without neglecting the autonomous nervous system hormonal mechanisms or the other adapting or deleterious bimolecular implications, the most relevant aspect is that, in this case, the cardiac structure does not provide contraction and ejection with sufficient systolic volume, and does not promote the adequate diastolic arrangements or both situations are not processed appropriately. And this heart dysfunction is closely associated with the heart geometric structural alterations. According to this statement, the aggregation of CF diagnostic stratification is mentioned, not only based on clinical symptom classes but also associated with structural dysfunction stages (A, B and D) proposed by the *Sociedade Brasileira de Cardiologia *[3] and the *American College of Cardiology/American Heart Association *[4] the guidelines for the CF diagnosis and clinical care. This proposition suggests all the manners of incorporating information on etiology data, physiopathology, geometric structure and heart function which range from an absent situation of structural heart alteration (phase A) to a terminal stage of myocardial dysfunction (phase D). At the same time, another recent highly interesting focus on the CF study involves the discussion of the actual importance in the search for information related to the left ventricular systolic and diastolic functions (LV) [5]. A direct consequence of this discussion is the conceptual division in CF with preserved contractile function and contractile dysfunction [6,7]. Recent population-based studies have shown an about equal prevalence of both with a higher mortality rate in the systolic dysfunction group, however, with similar indices in terms of morbidity [8]. Therefore, a new conception if CF emerged with the need for a conceptual definition based on the analysis of the LV systolic and diastolic function generating a dynamic and constant discussion on the method of cardiac functional assessment, the use of LV geometry additional data, the left atrium and direct chamber functions as well as the functional capacity indices. The complexity of the CF diagnostic clinical approach has increased in the last decade; on the other hand, with the advent of many heart evaluation indices, there is an increasing need to define which actions are really effective, the signified levels, its effect size and the cost which they represent.

## Diastolic function evaluation in cardiac failure patients

The LV diastolic function can be measured by estimate or index direct measurement through various cardiovascular imaging methods: radioisotopic ventriculography, heart catheterization, and magnetic resonance imaging, however, the echocardiographic-Doppler study is the most applied tool, due to its high feasibility of transmitral Doppler indices, almost universal availability and low cost [9].

## Echocardiography by transmitral pulsed Doppler and pulmonary veins: a description of classical qualitative patterns and method limitations

Since 1980, the pulsed Doppler technique for the left ventricular analysis allows the diastole study in a noninvasive form, initially in animals then in human beings [10]. The information obtained with the technique permits standardization within acceptable variations of what the normal and the alternate diastole are in terms of echocardiography.

**Figure 1:**
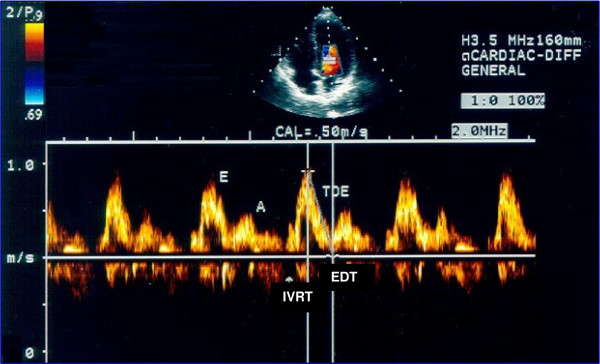
**LV diastolic indices measured by transmitral Doppler**. E: LV early filling flow velocity; A: late filling flow velocity; EDT: E-wave deceleration time; IVRT: LV isovolumic myocardial relaxation time.

Various studies have used the transmitral Doppler (TD) recognizing normal and alternate diastole patterns. According to the technical point of view, the TD analyzes the blood flow by measuring the high frequency signals and the low amplitude of the blood cells [11]. The method proposes the following indices for the diastolic analysis: E-wave (early ventricular filling); A-wave (late ventricular filling); E/A ratio; isovolumic relaxation time (IVRT); E-wave peak velocity (E); E-wave deceleration time (EDT), A-wave duration (A-dur). The flow analysis by pulsed Doppler of the pulmonary veins allows the acquisition of additional indices: flow systolic peak (S) – divided in S1 an S2-flow diastolic peak (D), atrial reverse flow peak (AR) and duration rate of this reverse flow (AR-dur). Important information for the diastolic pattern characterization is obtained through a detailed analysis of the available parameter set, making use of the transmitral Doppler method and the pulmonary veins, as follows: (a) normal function: generally observed in youngsters; the early filling is dominant with E/A ratio > 1. There is a light predominance of peak D and AR and AR-dur minimal amplitude; deficient relaxation or relaxation deficit: there is a minor peak of early filling velocity; therefore, the E/A ratio is > 1. The IVTR and the EDT are higher. In pulmonary veins, the D velocity according to the E-wave is diminished and compensated by the systolic S flow. The AR and the AR-dur usually remain unchanged but can increase according to the elevation of the LV final diastolic pressure. The pattern is related to ischemia, hypertrophy or even to infiltrative cardiomyopathies in initial stage; (b) *Pseudonormal *pattern: it presents an E/A > 1 ratio as the normal pattern; however, it reflects a velocity increase of the E-wave flow secondary to the pressure elevation in the left atrium, a relaxation deficit and an initial decrease of the LV compliance. The IVTR is diminished due to the higher initial transmitral gradient. The pulmonary venous flow presents abnormal D velocity predominance as this one relates with the increase of the early mitral flow (E-wave). This situation represents an ischemia dysfunction progression, hypertensive or LV overload eventually becoming difficult to differentiate in relation to the real normal pattern; (c) restrictive pattern: the velocity of the early diastolic filling is increased which results in E/A ratio > 2 and in an IVTR and EDT decrease. In this case, the rapid blood flow to the less complacent ventricle results in a rapid elevation of the LV filling pressure, supplanting the atrial pressure which could also cause ventricle-atrial regurgitation in the diastolic phase. As it was observed in the previous pattern, there is here a pulmonary venous flow diastolic increase which corresponds to the transmitral E-wave elevation. The AR and the AR-dur are increased and they keep a positive correlation with the LV final diastolic pressure. The restrictive pattern is related to advanced stages of heart failure and with the worst clinical prognosis (Figure 1).

The TMD index contribution to the CF diagnostic and prognostic measurement is undeniable [12-15], even though the method is questionable under the technical point of view. The difficulties and limitations are based on the variability in different clinical and haemodynamic conditions. The effects of the left atrial pressure variation on the LV preload different degrees eventually produce a *pseudonormalization *or even a restrictive pattern type; the heart rate elevation leads to the velocity alterations and often to the spectral Doppler wave fusion with a consequent loss of the qualification analysis of the ratio between the waves. Meanwhile, the LV preload variable status can also set alterations inversely proportional in the TMD indices. It is reasonable, however, that this method is complemented with others for a comprehensive ventricular filling function analysis [16].

## Pulsed tissue Doppler contribution for the diastolic measurement

The tissue pulsed Doppler (TPD) consists of a modality linked with the echocardiography by Doppler which allows the estimate of the myocardial displacement velocity during the cardiac cycle analysing signals of low frequency and high amplitude produced by the myocardial tissue [11].

**Figure 2:**
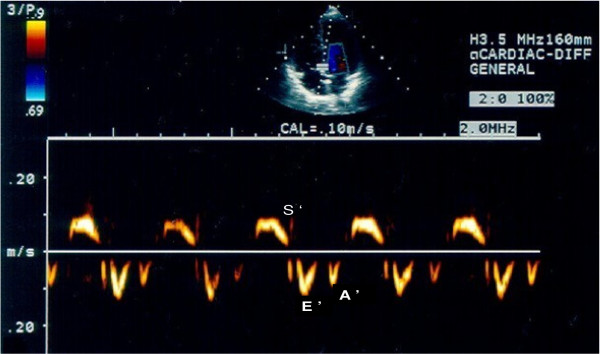
**LV diastolic pattern by tissue Doppler**. S': LV systolic myocardial displacement velocity; E': LV early diastolic displacement velocity; A: LV late diastolic displacement velocity.

**Figure 3:**
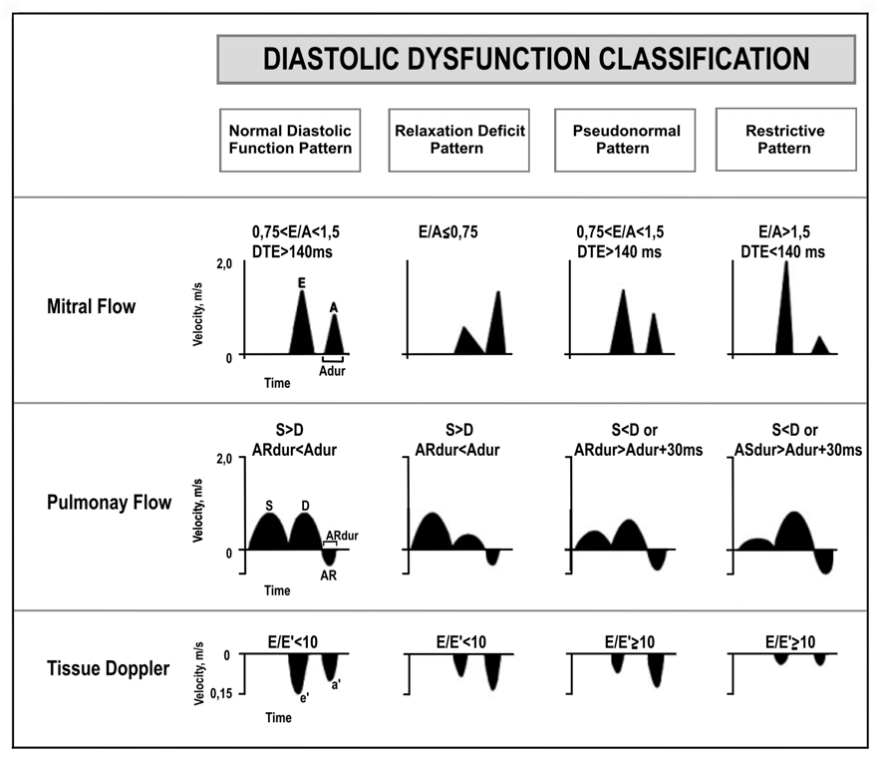
**LV diastolic patterns measured by transmitral Doppler of the pulmonary flow and tissue Doppler**. E/A ratio between the LV early filling flow E velocity and late flow A measured by transmitral Doppler EDT: E-wave deceleration time; S: pulmonary flow systolic velocity peak; D: pulmonary flow diastolic velocity peak; RA: retrograde atrial velocity peak; RA-dur: retrograde atrial flow duration; ms: milliseconds; E/E: ratio between the early filling flow E velocity measured by transmitral Doppler and the LV early diastolic myocardial displacement E' velocity measured by tissue Doppler.

The myocardial velocity phenomenon can be analyzed by three models: bidimensional coded by color, unidimensional coded by color, and the more commonly used, the pulsed spectral mode, with which the systolic myocardial displacement S', and the E' and A' waves can be respectively registered (Figures 2 and 3).

The tissue Doppler E' velocity index is primarily influenced by left atrial pressure, left ventricle relaxation and left ventricle systolic pressure in order of decreasing significance [16], and for its procedure, this and the other indices are taken with the volume-sample placement in the myocardial region next to the mitral valve ring in the LV septal and lateral wall or in any other myocardial segment which will be measured [17]. This possibility in various myocardial segments confers the "segmental diastolic" information acquisition property, which can also be useful in myocardial ischemia investigation and in the ventricular dissynchronism analysis [18].

## Which evidences do validate the tissue Doppler index addition?

**Figure 4:**
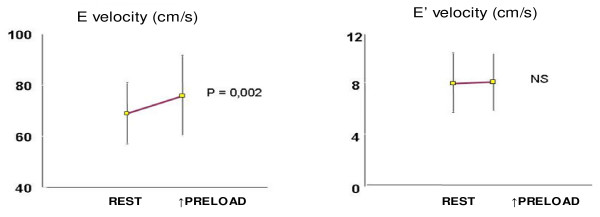
TMD and TPD indices in basal condition and preload increase.

The TMD index susceptibility, in relation to the LV preload variations, represents this method main disadvantage for the diastolic analysis in a global manner. The TPD indices contribute to a less susceptible measurement to the preload variation. In a recent study performed by our research group, we have observed that in the hypertensive diastolic measurement, there was not significant variation of E' index with the bedside noninvasive maneuvers that induces the preload elevation (E' = 8.2 ± 2.3 cm/s × E' = 8.3 ± 2.2 cm/s, P = NS), while the TMD index E has varied significantly (E = 68.9 ± 11.9 cm/s × E = 75.8 ± 15.7 cm/s), (Figure 4). This property could be very helpful in the differentiation between the normal and *pseudonormalized *patterns [19,20].

## Mitral Doppler and tissue pulsed Doppler indices in basal condition and elevation of preload

In the invasive study model, Sohn et al [21], already showed the advantages of this method in relation to the transmitral flow summarized in the preload reduced influence in the E' variable obtained by TPD. While analysing 20 patients with diastolic dysfunction by altered relaxation – detected by transmitral Doppler – an abnormal relaxation diastolic pattern change to *pseudonormalized *was observed after the infusion of 500 to 700 ml of saline physiological solution (0.9% NaCl concentration), but the TPD diastolic pattern analysis did not show any significant alteration (E' = 5.3 ± 1.2 cm/s × E' = 5.7 ± 1.4 cm/s, P = NS).

This TPD index behavior was partially questioned in a recent publication by Hsiao et al. [22] in which the authors have studied 81 patients in hemodialysis without segmental contractile alterations, stratified according to the weight loss post-dialysis and observed preload independence when the dialytic loss was less than 2 kg. When the loss was higher, the TPD varied significantly. Even so, it is consensual the fact that the technique is less pre-load dependent than the traditional DTM indices and that it adds significant information to the diastolic evaluation. However, in a population similar to the one referred above, and original study has shown in counterpoint to the latter, that there was no significant variation in the TPD, E', A', and E'/A' indices before preload variation by lower member sustained elevation imposed on patients of all ages, one hour after the hemodialysis, while the MTD, E, A and E/A indices varied significantly [23]. It is extremely important to emphasize that the E' index reflects the early ventricular relaxation and it has also been validated against the *Tau *index (time constant isovolumic relaxation), considered the gold-standard for LV myocardial relaxation, according to Sohn et al. [21]. In this study, the authors have obtained a correlation between E' and *Tau *(r = -0.56, P < 0.01) in 38 volunteers with relaxation abnormalities. Recently, an important study has been published by Nagueh et al. [24] in which there were an attempt to relate MTD and TPD data with pulmonary capillary pressure measures through pulmonary artery catheterization of 60 volunteers.

When both methods (MTD and TPD) were used in E/E' ration, a strong and statistically significant correlation was obtained with the capillary pressure measured by catheter (r = 0.87, P < 0.001). Besides, the study allowed the verification of patients with E/E' ration > 10 predicting a pulmonary capillary pressure > 15 mmHg with 97% sensibility and 78% specificity, and this study has placed the E/E' ratio in a quite promising position, as a diastolic index, stimulating many other subsequent studies. Ommen et al. [25], while analysing 150 consecutive patients set out for cardiac catheterization and submitting them to an echocardiograph evaluation MTD and DPT concomitantly to the intraventricular pressure measurement, have verified a significant correlation of the E/E' ratio with the measured LV diastolic average pressure (r = 0.63), and E/E' > 15 which was associated with a ventricular pressure above 15 mmHg with 86% specificity (with a 64% positive predictable value). In 2001, Nagueh et al. [26] identified a strong correlation with the *Tau *index (r = 0.83, P < 0.001) and the pressure negative variation over the LV time variation (-dP/dt) (r = 0.8, P < 0.001), another parameter used in the LV relaxation assessment, aiming at better studying myocardial diastolic velocity determinants by Doppler tissue in animal sample with left atrium (LA) and LV pressure catheterization and measurement. For the first time in this study, the LA hemodynamic data are investigated invasively in relation to the TPD indices presenting a significant association between them: the A' late diastolic velocity was significantly correlated with the dP/dt (r = 0.67, P = 0 < 0.01), and with the LA relaxation index (r = 0.67, P < 0.01). Additionally, there was an inverse and significant correlation with the LA final diastolic pressure, which also reflects indirectly the LA hemodynamic behavior (r = 0.53, P < 0.001). Another concern, regarding the diastolic evaluation in patients with probable CF, is the lack of an isolated TMD index reproducibility when evaluated in an altered rhythmic situation, as is the case of atrial fibrillation or sinus tachycardia. Some studies, including the TPD indices, have approached this problem recently. In 1999, Sohn et al. [27] studied LV invasive hemodynamic measurement data concomitantly with echocardiography with TMD and TPD in 27 patients with atrial fibrillation of non-rheumatic etiology. The E/E' ratio had a positive correlation with the left ventricular filling pressure (r = 0.79, P < 0.001). When this index presented values ≥ 11 it allowed the prediction of LV high filling pressure (≥ 15 mmHg), with a 75% sensibility and 93% specificity. Another study by Nagueh et al. [28], while evaluating 100 patients with a heart rate ≥ 100 beats/minute, described a positive and significant correlation between the E/E' ratio and the pulmonary capillary pressure value (r = 0.86, P < 0.01). An equation, was also proposed by this author and has shown a strong correlation with the pulmonary capillary pressure measured by catheterization (r = 0.91, P < 0.05).

The evidences of such transversal study commented here for small populations, and some of them not using the real gold-standard for comparison, can still be questioned regarding its validity. However, when information arises from some recent data from a group of people added, involving Doppler indices in patients with CF, there is an awareness of the prognostic prediction power in mortality regarding hard events in patient groups with chronic atrial fibrillation [29], survival in the post myocardial infarct period [30], or with systolic heart failure, as in the ADEPT study [31]. Finally, it is important to emphasize that the Doppler tissue analysis does not aggregate additional cost to the exam and the necessary time for the acquisition of such indices is not relevant.

## The LV remodeling evaluation: is it a landmark in the etiologic, diagnostic and prognostic investigation in patients with CF?

The LV remodeling is generated by mechanical, neurohumoral, and possibly genetic factors which alter the ventricular dimension, morphology and function. It can occur in response to the aggressions in many clinical conditions including severe or chronic ischaemia, cardiomyopathies, hypertension and valvular and infectious disease. In the structural alteration diagram, hypertrophy, myocyte loss and the interstitial fibrosis increase, compose the tissue physiopathologic aspect associated with a progressive dysfunction. In its conception, the CF cause and consequence precepts are superimposed [32], as the mechanisms responsible for its genesis can generate a retro feeding or vicious cycle, worsening the geometric pattern progressively. The elucidation of the mechanisms responsible for the prevention and/or remodeling process reversibility is one of the most important CF investigation areas. The interventions which have already showed positive impact on the survival rate have a favorable action on the myocardial remodeling process [33]. It can be observed with angiotensin conversion enzyme inhibited medication [34], beta-blockers [35], and even in more complex therapies like accessory circulatory support [36] and stem cell therapy, as proposed in the TOPCARE-AMI study [37]. These evidences confer to the remodeling indices the substitutive parameter epidemiologic distinction associated with strong clinical outcomes like mortality becoming an essential element to be analyzed in the clinical practice involving CF patients. A pertinent example is the possibility of a LV remodeling index, as the LV mass ratio index/corporal surface (IMVE) showing a ventricular hypertrophic remodeling pattern, to be able to signal a hypertensive etiology as the hypertrophy and the arterial hypertension are commonly associated, as suggested in the ICARE study [38]. The same geometry index can simultaneously quantify the severity level of a hypertensive cardiac disease and determine the prognosis in terms of cardiovascular mortality, general mortality and cerebral vascular accident. According to a study by Devereux et al. [39], in a group of 941 patients with left ventricular hypertrophy whose myocardial mass was measured by echocardiography followed up by a 4.8 year average period, regarding the major cardiovascular events at the end, there was an association between the lowest ventricular mass index (patients with anti-hypertensive treatment) and lower cardiovascular mortality rate (HR = 0.62; CI 95% 0.47–0.82; P = 0.001), lower cerebrovascular rate (HR = 0.76; CI 95% 0.60 – 0.96; P = 0.02) and lower mortality for all the causes (HR = 0.72; CI 95% 0.59 – 0.88; P = 0.002) So, it can be concluded that the information conferred by LV remodeling qualitative and quantitative analysis can be somehow important for the CF clinical approach and investigation.

## The LV diastolic function and remodeling

The study of the changes in the association in ventricular geometry with the worsening of specific diastolic stages – LV relaxation, and compliance or stiffness – represents an interesting focus. While still insisting on the association between SAH and ventricular dysfunction remodeling in a patient with CF, it is important to remember that, in this case, the hypertrophy works as an adaptive mechanism for the LV pressure overload involving the muscular and non muscular heart compartment. Various studies suggest the role of the contractile protein increase in the myocyte with a subsequent increase of this cell, and followed by the collagen matrix remodeling and the sustained growth in the deposition of this element in the interstitial space. The natural history of this process is the worsening of the myocardium active relaxation and the increase of the chamber stiffness causing LV filling dysfunction [40]. Studies on LV hypertrophy using biopsy and hemodynamic data suggest that the existent myocardial fibrosis contribute to the diastolic pressure increase and alters the chamber distensibility when the amount of fibrosis increases more than 15 to 20% [41]. Characterization of pathological hypertrophy is important as a diastolic dysfunction genesis. In a physiological hypertrophy situation, in response to physical exercises, there is the proportionality of the vascular, interstitial and muscular components. This tissue homogeneity in hypertrophy observed in athletes is not associated with the diastolic pattern dysfunction, but with an adaptation of the relaxation myocardial velocity increase and normal profile maintenance of the LV late and early filling flow ratio [42]. In the pathological hypertrophy there is a growth of the nonmuscular component of the myocardium with intercompartmental proportionality loss. While investigating this sub-item, Villari et al. [43] have studied patients with hypertrophy due to aortic stenosis separating them in three different groups according to the myocardial biopsy results: the first group with collagen total volume fraction increase but without collagen fibers disorganization pattern; the second one, with fiber disorganization with no collagen volume fraction increase, and the third one with fiber disorganization and collagen increase. The author has observed an exponential ratio between the collagen volume and the constant ventricular stiffness. This correlation was not observed in group 2, which presented a collagen normal volume and a highly constant LV stiffness. This discrepancy can be explained by the differences in the collagen fibrillar distribution and architecture and indicates that not only the collagen amount but also its distribution and configuration determine the LV myocardial elastic property role.

The hypertrophy relation with LV relaxation alteration has also been investigated and deserves some special consideration. In relation to patients with systemic arterial hypertension, Oki et al. [44] have studied the diastolic dysfunction by echocardiography with Doppler using two groups of individuals: fifty hypertensive patients and thirty six normal volunteers, and submitted them to TMD and TPD analysis. The results showed a myocardial relaxation velocity significantly lower in the hypertensive group. Besides, there was a significant correlation (r = 0.80, P < 0.001) between the myocardium early displacement velocity and the *Tau *index, rectifying previous findings [21]. The classification of the hypertrophic LV geometric configuration types in remodeling stages have also demonstrated clinical relevance and preliminary echocardiographic studies have already shown some worsening correlation degree of the ventricular diastolic function with its geometric alteration degree. In the LIFE study analysis, Wachtell et al. [45] have found an isometric relaxation time, as well as a LV relaxation measurement, significantly higher in hypertensive patients with a higher geometric mass pattern. In the following year, a Chinese group published in the *Hypertension Research *[46] a study which approaches this question more deeply. Analysing a group with 117 hypertensive patients and 45 normal volunteers with echocardiography to obtain mass index data and parietal thickness ratio, which were used to characterize the normal geometry (N), concentric remodeling (CR), concentric hypertrophy (CH) and eccentric hypertrophy (EH) groups according to Ganau et al. [47], and measuring its relation with the Doppler diastolic patterns (MTD only), this author has observed a progressive worsening of the diastolic indices as the hypertrophy patterns became more severe. There was a significant difference of these indices especially in the groups with CH and EH in relation to the ones with normal geometry. Some recent findings were demonstrated recently in a substudy of a larger project named *HyperGEN Study *[48] with 1,384 hypertensive individuals, in which LV filling pattern analysis by Doppler and by TMD (E/A, IVRT and EDT) was compared with four different geometry patterns as said previously. The abnormal relaxation TMD pattern of the *European Society of Cardiology *[49] was observed in 20% of the patients. The E/A, and IVRT did not show an abnormal relaxation pattern when compared to the presence of LV hypertrophy, but they indicated such behavior when the hypertrophy was concentric (P < 0.001). The application of logistic regression test has revealed that the chances for an abnormal relaxation pattern to occur were two to three times higher in CH in relation to the LV normal geometric pattern. These last two studies have rectified the previous propositions and seem to have validity for an universe of hypertensive patients with remodeling and with no contractile and filling dysfunction severity, however, the authors have worked only with TMD diastolic parameters, a fact which was left out by the authors of the previous study and can limit the evidence of the findings as it was mentioned earlier, since the behavior of these indices is affected by the LV preload which determines a non linear distribution character, but ellipsoid in relation to the ventricular filling pressure value. Clarifying, an E/A ratio higher in relation to another one does not necessarily mean that in the second patient a better diastolic status would be expected. By analysing all the evidence context of the LV remodeling relation with its diastolic function, however, one cannot conceive a disconnection of one with the other but a new perception when aggregating geometric structure and function seems more rational and adequate. Would this way of thinking be complete without adding information regarding the anatomy data and atrial function?

## The left atrium: pump, reservoir, conduct or a dam?

The discussion on the left atrial physiology must be preceded by a brief review of the liquid dynamic concepts and based on this, a combination with the basic notions of its wall contractility, compliance and relaxation biological properties. The LA is a functioning chamber more complex than it seems and it is not a LV simple appendix or a pulmonary vein final chamber but an structure with direct implication on the heart rhythm, on the pulmonary circulation and on the LV filling modulation, with a defined clinical importance and has been largely discussed in the real scenario in the heart failure syndrome, systemic arterial hypertension clinical context, and even in normal, athletic or sedentary patients [50]. Even though it is a multifunctional structure, its property used to contract and propel volume to the LV is always the one referred to when someone evaluates the LA participation in the cardiac cycle. However, the pump function has a discrete importance in patients with normal LV and its contraction function is more necessary in physical exercise situations. The amount of contribution of the atrial systole for the LV function is controversial, depending on the LV own function and the heart rate, and this will influence in the increase of the LV systolic and diastolic efficiency. In other words, the LA contraction acts like an auxiliary propeller which increases the ventricular systolic volume by the increase of the LV volume and diastolic pressure using the Frank-Starling mechanism. In pathological situations, the pump becomes of compensatory real importance in relaxation dysfunction states and/or ventricular compliance, maintaining a LV minimum filling volume and a normal LA pressure [51]. The reservoir function is also relevant. The chamber operates as storage at the end of the line for the pulmonary venous flow, a predominant factor for the generation of enough atrial pressure to produce a LA-LV gradient, a phenomenon which is necessary for the LV early filling when the repletion of 80% of the diastolic volume happens in normal conditions. Therefore, the greater the capacity to preserve volume, greater will be the energetic contribution for the atrial systole, considering the Frank-Starling mechanism one more time [50]. The reservoir mechanism by which the LA works is the function of the relaxation, the left atrial complacence and the LV longitudinal shortening. In the CF caused by ischaemia or mitral regurgitation, this reservoir can operate by accommodating volume and pressure, acting as a pulmonary circulation protector, at least for a certain time. At the very last, the LA acts as a simple flow conduct for the LV. The chamber assumes this propriety in two moments: an early phase, during the ventricular relaxation and mitral valve opening and in a later phase between the early ventricular filling and the late one, acting as a linking direct conduct between the pulmonary veins and the LV taking the advantage of the open mitral valve. In this last case, the flow happens due to the atrial-venous gradient difference and it only occurs in low heart rate situations [52]. The LA works predominantly as a conduct in opposing clinical situations for the normal individual, in which the reservoir function or the atrial contraction is not that important or in situations of extreme increase in the diastolic pressure and/or LV stiffness in which the atrial systole cannot contribute anymore to the ventricular filling due to the LV pressure/volume high relation. To conclude, it seems more rational to consider the reservoir properties, conduct and contraction as a LA unique function due to the much interdependence among them [52,53]. This function synthesizes the auxiliary pump important role for the LV and due to its protective action for the pulmonary circulation, from a regulative dam to pulmonary circulation.

## The left atrium and its interaction with the diastolic function and left ventricular remodeling: the evidences and its clinical applicability

The remodeling indices and the left atrial function have already been related to systemic arterial hypertension, age and a variety of other situations, and the interaction with the LV function represents an important focus of interest to understand the CF myocardial physiopathology. The left atrium contractile function is the most emphasized item when this issue is discussed – LA and the LV function. The awareness regarding the contribution of the atrial systole for the increase of the relation LV pressure/final diameter has opened new perspectives for the understanding of the geometry relation model, the left atrium function and its relation with the LV [54]. Kono et al. findings [55], in an animal sample of CF induced by coronary embolization, have demonstrated a significant LA increase dimension from the beginning to 33 weeks after the induced CF (2.4 ± 0.2 cm × 3.3 ± 0.3 cm, P < 0.01). The systolic function has also been affected and its shortening fraction has worsened in the period (22 ± 3% × 15 ± 2%, P < 0.01). Both alterations were associated with the significant worsening of the LV filling TMD pattern. The author has concluded that a LA dimension increase should represent a compensation for the LV early filling decrease in the induced CF. Additionally, the worsening of the shortening fraction would be a reflex of the LA contractile compensation early failure regarding the more adverse LV pressure and volume conditions. This publication is highly illustrative as it approaches a classical profile of the post-ischaemic CF and the results converge to an understanding of the prognosis power after AMI of the LA volume indices which have been demonstrated recently [56]. Another urging issue to be analysed, is the study of the LA geometric and functional index relationship with LV filling pressure measures. Appleton et al. [57] have investigated 70 patients with probable ischaemic heart disease submitted to cardiac catheterization and to an echocardiography evaluation with Doppler of the left atrium and the LV diastolic function. One of the objectives was to estimate the LV filling pressure using echocardiographic study with Doppler and the additional value of the dimension indices and the left atrium function in this prediction. The results have revealed a significant correlation between the average pulmonary capillary pressure and the LA volume (r = 0.70) and a 82% sensitivity with 92% specificity, so that the minimum left atrial volume > 40 ml could estimate an average pulmonary capillary pressure of 12 mmHg. This study reveals important homodynamic evidences, as the filling pressure can be considered an excellent comparison pattern and well correlated with the clinical symptoms, therefore being able to suggest clinical relevance for the atrial volume indices measured by echocardiography. Following the clinical approach, it is important to mention that systemic arterial hypertension represents one of the highest stimuli for heart structural disease.

However, what would be the correlation between the atrioventricular remodeling variables, LV myocardial dysfunction by pressure overload in heart adaptation initial stages to systemic arterial hypertension? In an attempt to clarify this question, a study by Tsioufis et al. [58] has shown in a group of 94 patients with primary SAH in stages I and II, comparing to 34 controls without hypertension, a higher index of the LV mass (105 g/m2 × 84 g/m2, P < 0.001), a larger LA diameter (39 mm × 36 mm, P < 0.001), and a bulkier LA (22 × 19 ml/m2, P < 0.05). In the hypertensive population, the LA volume index correlates significantly with the LV mass index, pulse pressure, systolic pressure level and natriuretic peptide. It is relevant to call the attention to the idea that hypertension is a dominant factor in such referred population of elderly people with their hearts insufficiently dependable to preserve contractile function. Another article published by a group from the *Wake Forest University *[59] has studied the volume relationship geometry and left atrial function in the diastolic and systolic CF population of 851 elderly subjects > 65 years. The authors have observed that the volume, the area and the linear dimensions were higher in the CF incident and more dominant groups in relation to the control group and have not seen differences during the follow up periods of groups with diastolic versus the systolic cardiac failure.

These findings emphasize the importance of the left atrium as an actor in CF patients independently of the case of depressed or preserved LV ejection fraction, validating the applicability of this parameter in patients in this age group with diastolic CF. In short, it is possible to state that there is some evidence support which points out to a clear idea of an interaction between the geometry data and the LV and LA function. The next step is to establish and to quantify the importance of all information for clinical handling. And, more recently, in agreement with this idea, many studies were published, transcending concepts obtained in a transversal way for studies of longitudinal follow up which emphasized this issue relevance. In at least four studies published in the last time, a left atrial index prognostic correlation could be observed. Tsang et al. [60] have observed that increased atrial indices presented a prognosis prediction value very significant for cardiovascular events in a population of 317 individuals in sinus rhythm, where the left atrium volume index has obtained the best performance (area under the ROC curve 0.71 for the LA volume index and 0.59 for the LA diameter). In another publication of the group from the *Mayo Clinic*, in which a cohort from Olmsted, Minnesota, USA was investigated with 1,375 elderly people (≥ 65 years of age) with preserved systolic function (≥ 50% of LV ejection fraction), it was identified that a left atrial volume ≥ 32 ml/m2 was an independent predictor for the first manifestation of CF (P < 0.001) in a follow up of 4.3 ± 2.7 years of age and a higher LA volume progression (8 ± 10 ml, P < 0.001) was observed in follow up of people who developed CF [61]. There are evidences pointing out that maintenance of the left atrial contraction function has a better prognosis. Echocardiographic data from 2,808 middle age individuals and elderly ones of the population-based study *The Strong Heart Study *[62] conducted in North-American Indians has revealed that the elevated LA systolic strength (90 percent) was associated with a higher rate of combined cardiovascular events even though with a modest effect proportion, but statistically important (HR = 1.33, CI 95%, 1.05 – 1.61; P = 0.021), besides being associated with geometric changes and heart functions. This was an original finding which has enriched the knowledge on the atrial function as the clinical prognosis marker in the primary prevention context of cardiac events among them the CF patients. Another publication involving the same group of people aimed at correlating the simple left atrial diameter measure with the incidence of combined cardiovascular events. In this analysis, an increase in the atrial diameter (4.2 cm for men and 3.8 cm for women) was an independent risk predictor for the first episode of combined cardiovascular events (HR = 1.57, CI 95% = 1.17 – 2, P = 0.002). When analysing data from this later study, someone may come to the conclusion that the abnormal LA has identified a population with more prevalent heart dysfunction, with LV relaxation abnormalities and a most elevated filling pressure level. Besides, changes of the LA geometry joined the production of natriuretic peptide and have a direct relationship with atrial fibrillation genesis and other arrhythmia and these ones with the formation of embolus inducing cerebral ischaemia [63]. Studies also suggest that the echocardiographic variables of the LA may predict a higher susceptibility of such patients and should be the reason or basis for more aggressive approaches of a primary prevention [64]. As in hypertensive groups, some previous works have shown an atrial growth reversion with anti-hypertensive adequate therapy, probably, so, the evidence of the atrium data use in the diastolic heart function evaluation is clear, and despite the LA volume index superiority, the simple diameter measure is also associated with the cardiovascular prognosis.

## Atrioventricular remodeling and diastole: a combined analysis

**Figure 5:**
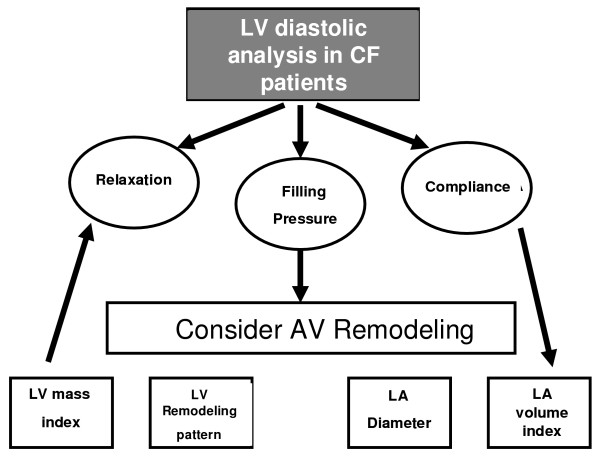
Left ventricle diastolic analysis in CF patients.

The diagnostic evaluation of the patient with a suspicion of CF is not always simple, mainly when there is a normal or slightly altered LV ejection fraction, since this is the case in half of the CF cases. The echocardiographic tools for diastolic evaluation, despite the improvement already attained, sometimes are not enough if they are analyzed separately (Figure 5). The LV and LA remodeling data already fulfill the gaps left by Doppler and allow a more complete diagnosis characterization, gathering prognosis information. The consideration of validated indices like the LV mass index, the LV eccentric or concentric hypertrophy pattern, the left atrial volume index or the LA diameter in the diagnosis analysis can represent a fine adjustment fully tuned up with the tendency mentioned in clinical studies.

## Competing interests

The authors declare that they have no competing interests.

## Authors' contributions

This review article is part of the doctoral thesis of the author LCD under orientation of MRT, his advisor; LCB and IK participated in the examining commission of the author (in order to obtain his degree) and helped much in the process of writing and reviewing o this manuscript. All authors read and approved the final manuscript.

## Funding

The authors declare that they have no source(s) of funding.
